# Insight into the Unfolding Properties of Chd64, a Small, Single Domain Protein with a Globular Core and Disordered Tails

**DOI:** 10.1371/journal.pone.0137074

**Published:** 2015-09-01

**Authors:** Aneta Tarczewska, Małgorzata Kozłowska, Piotr Dobryszycki, Magdalena Kaus-Drobek, Michał Dadlez, Andrzej Ożyhar

**Affiliations:** 1 Department of Biochemistry, Faculty of Chemistry, Wrocław University of Technology, Wybrzeże Wyspiańskiego 27, 50-370, Wrocław, Poland; 2 Institute of Biochemistry and Biophysics, Polish Academy of Science, Pawińskiego 5a, 02–106, Warsaw, Poland; CNR, ITALY

## Abstract

Two major lipophilic hormones, 20-hydroxyecdysone (20E) and juvenile hormone (JH), govern insect development and growth. While the mode of action of 20E is well understood, some understanding of JH-dependent signalling has been attained only in the past few years, and the crosstalk of the two hormonal pathways remains unknown. Two proteins, the calponin-like Chd64 and immunophilin FKBP39 proteins, have recently been found to play pivotal roles in the formation of dynamic, multiprotein complex that cross-links these two signalling pathways. However, the molecular mechanism of the interaction remains unexplored. The aim of this work was to determine structural elements of Chd64 to provide an understanding of molecular basis of multiple interactions. We analysed Chd64 in two unrelated insect species, *Drosophila melanogaster* (DmChd64) and *Tribolium castaneum* (TcChd64). Using hydrogen-deuterium exchange mass spectrometry (HDX-MS), we showed that both Chd64 proteins have disordered tails that outflank the globular core. The folds of the globular cores of both Chd64 resemble the calponin homology (CH) domain previously resolved by crystallography. Monitoring the unfolding of DmChd64 and TcChd64 by far-ultraviolet (UV) circular dichroism (CD) spectroscopy, fluorescence spectroscopy and size-exclusion chromatography (SEC) revealed a highly complex process. Chd64 unfolds and forms of a molten globule (MG)—like intermediate state. Furthermore, our data indicate that in some conditions, Chd64 may exists in discrete structural forms, indicating that the protein is pliable and capable of easily acquiring different conformations. The plasticity of Chd64 and the existence of terminal intrinsically disordered regions (IDRs) may be crucial for multiple interactions with many partners.

## Introduction

Insect development and growth are governed by two major lipophilic hormones, a steroid, 20-hydroxyecdysone (20E) and a sesquiterpenoid, juvenile hormone (JH). 20E controls the initiation of molting, and its action is modulated by JH [1]. Whereas the mode of action of 20E is well known, the biological mechanism of gene regulation by JH is still unexplored [2,3], and little is known about the crosstalk between the two signalling pathways. Li *et al*. [4] recently identified a common 29-nucleotide JH-response element (JHRE) in the promoter regions of some JH-regulated genes in *Drosophila melanogaster* L57 and *Apis mellifera* brain cells and its binding proteins–the 21 kDa calponin-like protein (Chd64) and the 39 kDa FK506-binding protein (FKBP39). Chd64 and FKBP39 not only bind to JHRE but also interact with other nuclear proteins, including ecdysone receptor (EcR), ultraspiracle (Usp) and methoprene-tolerant (Met). Consequently, a model in which Chd64 and FKBP39 are a part of the multiprotein complex that mediates crosstalk between JH and 20E has been proposed [4]. The model represents an advance in our understanding of how factors involved in 20E and JH signal transduction have the ability to interact with each other, and which factors may be particularly important for the regulation of larval moulting and metamorphosis by JH. However, little is known about the structural properties that account for multiple interactions of Chd64 and FKBP39 with diverse protein partners and DNA. In our studies, we attempt to reveal structural features that may be important or necessary for the formation of a dynamic complex that cross-links hormonal signalling pathways. Here, we focus on Chd64 from *D*. *melanogaster* (DmChd64) and *Tribolium castaneum* (TcChd64). *Coleoptera* (*Tribolium*) is considered to occupy a basal phylogenetic position, whereas *Diptera* (*Drosophila*) is one of the most evolutionarily advanced insect genera. Both species show a classical developmental response to JH. Chd64 belongs to a family of calponin homology (CH) domain-containing proteins. The CH domain is a highly conserved protein module found in metazoans. CH domain-containing proteins vary in terms of the number of CH repeats and the existence of other defined motifs [5,6]. Interestingly, proteins containing only one CH domain are involved in either muscle contraction or signal transduction [5]. However, the structural features that enable the CH domain to perform such diverse functions, particularly regulatory functions, are not well understood [5–7]. It was previously shown that calponin-like Chd64 from *D*. *melanogaster* and *T*. *castaneum* are partly disordered; however, the extent and position of this disorder have not been defined [8]. Intrinsically disordered proteins (IDPs) and intrinsically disordered regions (IDRs) have been the focus of recent structure-function relationship studies [9]. Intrinsic disorder (ID) represents a new quality of proteins. This highly flexible and malleable structural state offers many advantages over ordered proteins; for example, in so-called one-to-many signalling, IDRs participate in different interactions with diverse partners [10,11].

This study was performed to identify structural features of Chd64 that may be important or necessary for the formation of a dynamic complex that cross-links hormonal signalling pathways. Hydrogen deuterium exchange mass spectrometry (HDX-MS) results revealed that two homologous proteins, DmChd64 and TcChd64, possess a dual structural nature (i.e., they comprise a globular core and disordered tails). Terminal IDRs may be critical for regulatory functioning because the disorder permits interactions with different proteins. Unfolding studies performed using a range of biochemical methods revealed that the native-to-unfolded transition proceeds through an intermediate state, which resembles molten globule (MG). Moreover, in some conditions, Chd64 exists in discrete conformational states, indicating that Chd64 molecules are pliable. This plasticity and the existence of terminal IDRs may serve as platforms for multiple interactions between Chd64 and various partners and may form the foundation for their regulatory functions.

## Materials and Methods

### Chemicals

Buffer A comprised 50 mM Na_2_HPO_4_, and 150 mM NaCl, pH 7.0, buffer B was 2 M glycine-HCl, pH 2.5, buffer C comprised 50 mM Na_2_HPO_4_, 600 mM NaCl, pH 7, buffer D was .50 mM Na_2_HPO_4_, 300 mM NaCl, pH 7 and buffer E comprised 100 mM Tris, 150 mM NaCl, pH 7.8.

All buffers were prepared at 24°C.

### Purification of DmChd64 and TcChd64

The purification of DmChd64 and TcChd64 was performed as previously described [8]. Briefly, the proteins were expressed in *Escherichia coli strain* BL21(DE3)pLysS (Novagen) growing on TB medium supplemented with 35 μg/ml chloramphenicol and 50 μg/ml carbenicilin. The cells were lysed by thawing at 25°C water bath and freezing twice and supplemented with an appropriate volume of PMSF and β-mercaptoethanol to a final concentration of 0.2 mg/ml and 1 mM, respectively. Then, DNase I and RNase A were added to the final concentration of 10 μg/ml of each enzyme and the lysates were incubated on ice until there was a loss of viscosity followed by 1 h centrifugation at 17 500 x g at 4°C. The first step of purification procedure for DmChd64 and TcChd64 was the same. The soluble fraction was collected and purified using immobilised metal ion affinity chromatography (IMAC). The cell lysate was incubated for 1h at 4°C, 40 rpm with 1200 μl of Co^2+-^TALON resin (Clontech) equilibrated with 5 ml of buffers C (DmChd64) or D (TcChd64). Subsequently, the resin was transferred into a Tricorn 5/50 column (Amersham Biosciences) and connected to the ÄKTAexplorer (Amersham Biosciences) system operated at 0.5 ml/min at room temperature. The column was washed with at least 5 ml of buffers C or D and then with at least 5 ml of a 10% gradient of buffer C or D supplemented with 250 mM imidazole. Finally the protein was eluted with 5 ml of appropriate buffers supplemented with 250 mM imidazole, collected and combined. For DmChd64, the second step of purification was affinity chromatography based on the Strep II-tag on the N-terminus of the protein. The solution containing DmChd64 obtained from the first step of purification was concentrated to a volume of 0.5 ml and applied to the Strep-Trap HP (Amersham Biosciences) column equilibrated with buffer E. The column was washed with 5 ml of buffer E and the fusion protein was eluted with 3 ml of buffer E supplemented with 2.5 mM desthiobiotin. After affinity chromatography gel filtration was used for both proteins. Fractions containing Chd64 were concentrated to a volume of 0.5 ml and injected in to a single Superdex 200 10/300 GL (Amersham Biosciences) equilibrated with buffer A and two tandem Superdex 75 10/300 GL (Amersham Biosciences) columns for DmChd64 and TcChd64 respectively. Fractions containing pure recombinant proteins were collected, combined, aliquoted and stored at -80°C.

### Hydrogen-deuterium exchange mass spectrometry (HDX-MS)

HDX-MS studies were performed as previously described [12] with some modifications. To determine the sequence coverage of DmChd64 and TcChd64 proteins, 5 μl of each 100 μM protein stock was diluted 10-fold by adding 45 μl of H_2_O reaction buffer (buffer A). The sample was then acidified by mixing with 10 μl of H_2_O quench buffer (buffer B) and subjected to immobilized pepsin column (Porozyme; ABI), using 0.07% formic acid in water as the mobile phase (flow rate 200 μl/min). Digested peptides were trapped on ACQUITY BEH C18 VanGuard Pre-column, (Waters) and then directed onto a reverse-phase Acquity UPLC BEH C18 column (Waters) with a 6–40% gradient of acetonitrile in 0.1% formic acid at 40 μl/min.r. All fluidics, valves, and columns were maintained at 0.5°C with a HDX Manager, only the pepsin columnwas maintained at 13°C inside of the temperature-controlled digestion compartment of the HDX Manager. The C18 column outlet was directly coupled with the SYNAPT G2 HDMS ion source (Waters). Peptides were identified using ProteinLynx Global Server software (PLGS, Waters). Then, the set of identified peptides was loaded into the DynamX 2.0 program (Waters).

HDX experiments were carried out as described above, but H_2_O was replaced with D_2_O. After mixing 5 μl of protein stock (here 60 μM) with 45 μl of D_2_O reaction buffer, the exchange reactions were carried out for 10 seconds at room temperature. The exchange was decelerated by reducing the pH; the pH was reduced by adding the reaction mixture to the quench buffer, which was cooled on ice. The sample was then incubated for 2 min on ice and then immediately injected and analysed by MS in Ion Mobility mode. Two control experiments were conducted to account for in- and out-exchange artefacts. Minimum exchange (IN control) was prepared by acidifying 45 μl D_2_O Reaction buffer upon addition of 10 μl of quench buffer, then protein (5 μl) was added into the sample. For the out-exchange experiment (OUT control), 5 μl of protein stock was mixed with 45 μl of D_2_O reaction buffer, incubated overnight, and then mixed with quench buffer. The control samples were analysed as other samples. The deuteration level of the IN control was denoted as 0% exchange (*M*_*ex*_0), whereas the OUT control as a 100% exchange (*M*_*ex*_100).

### HDX-MS data analysis

Deuteration levels for each peptide resulting from HDX were calculated with DynamX 2.0 software based on the peptic peptide list that was obtained using PLGS software and further filtered in DynamX 2.0. The final data were exported into Excel (Microsoft Office) for analysis. The percentage of deuteration was calculated using a formula (Eq 1) that considers the minimum (*M*_*ex*_0) and maximum (*M*_*ex*_100) exchange of a given peptide:

$$Deuteration\;\left(\%\right)=\frac{\left(M_{ex}-M_{ex}0\right)}{\left(M_{ex}100-M_{ex}0\right)}\times 100\%$$ (Eq 1)

Error bars for the deuteration percentage are represented by standard deviations of three independent experiments.

### Circular dichroism spectroscopy

Circular dichroism (CD) spectra were recorded using a JASCO J-815 CD spectropolarimeter (Jasco Inc., USA) equipped with a Jasco Peltier-type temperature controller (CDF-426S/15). Measurements were carried out in 1 mm path-length quartz cuvettes using DmChd64 and TcChd64 proteins at a concentration of 10 μM. The spectra were collected in a spectral range of 190–260 nm. All unfolding experiments were performed based on preliminary kinetic studies (data not shown) to find optimal conditions, especially the time of protein incubation with GdmCl. For the unfolding studies concentrated DmChd64 and TcChd64 in buffer A were diluted with buffer A supplemented with GdmCl to obtained 10 uM protein with appropriate concentration of GdmCl. The proteins were incubated on ice for 1 h prior the measurement. Reversibility of the process was monitored following an overnight dialysis of DmChd64 and TcChd64 at 6.0 M GdmCl against buffer A and proteins were concentrated to 10 uM. The final spectra were produced from an average of five measurements at a scanning speed of 20 nm/min at 20°C. All spectra were corrected for the contribution of the buffer, and all CD data were converted to molar residual ellipticity units.

### Fluorescence measurements

Fluorescence measurements were performed at room temperature using the Fluorolog-3-21 Horiba fluorometer (Jobin Yvon Inc., France) in 115F-QS quartz cuvettes (Hellma, Germany). DmChd64 and TcChd64 were dissolved to a final concentration of 7 μM and 10 μM, respectively, in buffer A alone or in buffer A supplemented with an appropriate concentration of GdmCl. Proteins were incubated with GdmCl for 30 min at room temperature before the spectra were recorded. The reversibility of the process was monitored following an overnight dialysis of DmChd64 and TcChd64 at 3.0 M GdmCl against buffer A. Fluorescence spectra were recorded between 300 and 400 nm, with an excitation wavelength of 280 nm. Titration experiments were conducted using a Microlab 500 automatic titrator (Hamilton, USA). Changes in the fluorescence intensities of the titrated proteins with GdmCl were corrected for the dilution effect and buffer contribution.

### Analytical size-exclusion chromatography

As described in details previously [8], analytical size-exclusion chromatography (SEC) was performed on a Superdex 200 10/300 GL (Amersham Biosciences) column connected to an ÄKTAexplorer (Amersham Biosciences) system and operated at 0.5 ml/min at room temperature. Detection was achieved by monitoring UV absorbance at 280 nm. The column was equilibrated with buffer A and calibrated with a mixture of the following standard proteins: thyroglobulin (85 Å) [13], apoferritin (67 Å) [14], bovine serum albumin (35.5 Å) [13], ovalbumin (30,5 Å) [13], chymotrypsinogen (20.9 Å) [13], myoglobin (20.2 Å) [15] and cytochrome *c* (17 Å) [15]. The total column volume (V_t_) was 24 ml, and the column void volume (V_0_) was 7.83 ml, as determined using blue dextran. The elution volume (Ve) of each protein was used to calculate the gel phase distribution coefficients (K_AV_ factors) according to Eq 2 [16]:

$$K_{AV}=\frac{V_{e}-V_{0}}{V_{t}-V_{0}}$$ (Eq 2)

Stokes radius (R_s_) values plotted against the corresponding K_AV_ factors of each standard protein were fitted to the standard curve. Purified DmChd64 and TcChd64 were prepared in buffer A with an **appropriate** GdmCl concentration and incubated on ice to reach equilibrium. 200 ul of protein solutions at 0.5 mg/ml were injected onto a column equilibrated with a corresponding buffer. Similar to described above, unfolding experiments, the reversibility of the process was monitored following an overnight dialysis of DmChd64 and TcChd64 at 6 M GdmCl against buffer A. Solution containing Chd64 was concentrated to 0.5 mg/ml and injected onto the column equilibrated with buffer A. The R_s_ values of DmChd64 and TcChd64 for each GdmCl concentration were estimated from the standard curve (described above).

### Analysis of the GdmCl-induced conformational transition of DmChd64 and TcChd64 molecules

#### Two-state approximation

In all unfolding studies, the pre- and post-transition baselines were linearly extrapolated to general forms, *y*_*N*_ = *ax* + *b* and *y*_*D*_ = *cx* + *d*, respectively, where *x* is the GdmCl concentration. These lines represent the values of measured parameters (e.g., ellipticity at 222 nm for CD spectroscopy) for molecules in the native (N) state and for molecules following conformational transition, i.e., in the unfolded state (D), at the corresponding GdmCl concentration. The D form percentage was calculated for each GdmCl concentration using Eq 3 [17]:

$$\%D=\frac{y_{N}-y_{ex}}{y_{N}-y_{D}}\times 100\%$$ (Eq 3)

where *y*_*ex*_ represents the experimental value of the measured parameter.

#### Number of conformational states

An approach used by Uversky and Ptitsyn [18] in their β-lactamase unfolding studies was used to analyse the number of Chd64 conformational states induced by the denaturant. The fraction of native (*f*_*N*_) molecules was calculated from the fluorescence emission maximum using Eq 4:

$$f_{N}=\frac{y_{D}-y_{ex}}{y_{D}-y_{N}};$$ (Eq 4)

The fraction of unfolded (*f*_*U*_) molecules was calculated from the Rs (obtained by SEC) and the ellipticity at 222 nm using Eq 5:

$$f_{U}=\frac{y_{ex}-y_{N}}{y_{D}-y_{N}}.$$ (Eq 5)

In our case, *f*_*N*_ + *f*_*U*_ < 1, and the fraction of the intermediate state (*f*_*i*_) was defined using Eq 6:

$$f_{i}=1-\left(f_{N}+f_{U}\right).$$ (Eq 6)

## Results

### The Search for intrinsic disorder in DmChd64 and TcChd64 by hydrogen-deuterium exchange mass spectrometry

**Fig 1:**
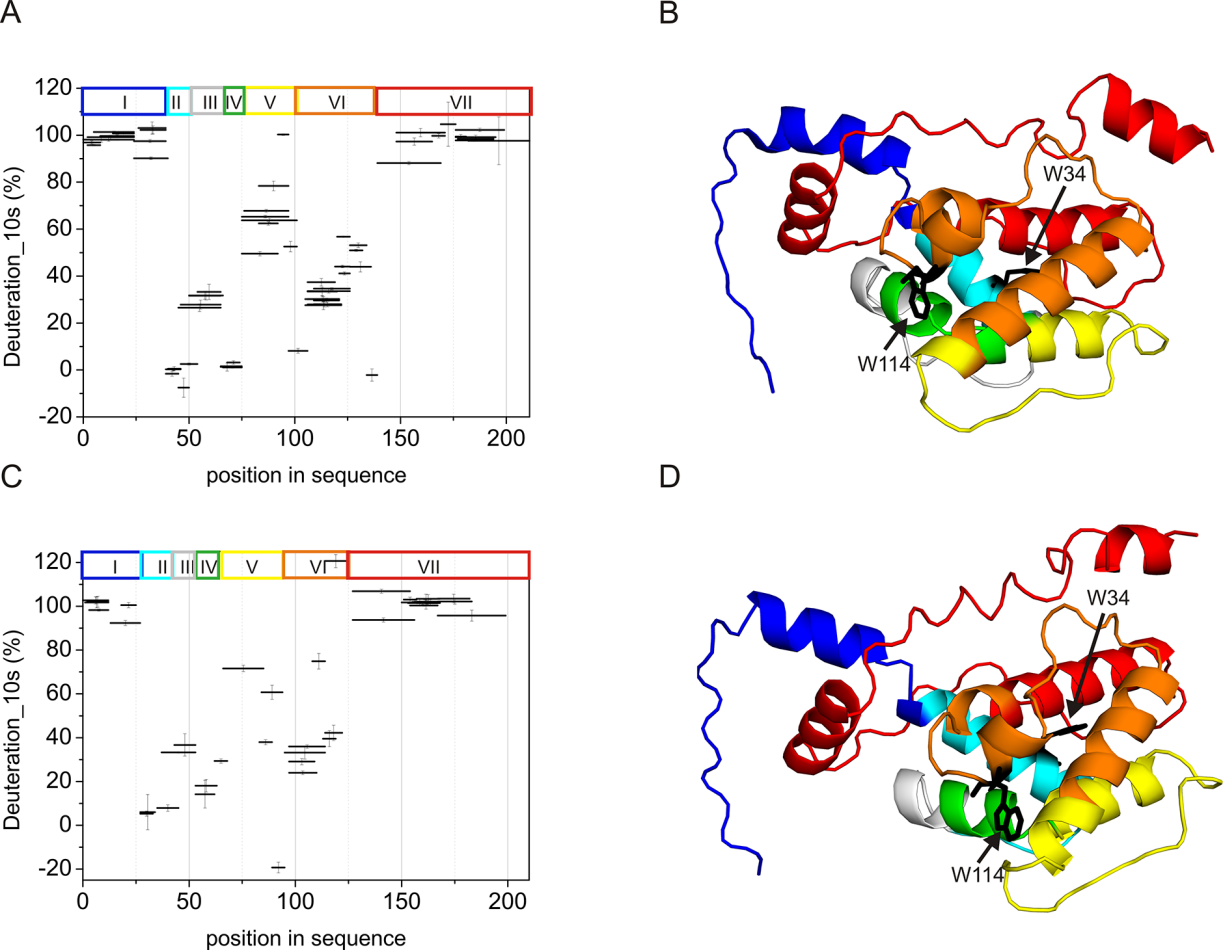
Hydrogen-deuterium exchange of DmChd64 and TcChd64. (A), (C) The deuteration percentage of peptic fragments from DmChd64 (A) and TcChd64 (C) in an exchange time of 10 s. The positioning of peptides in the sequence is shown along the horizontal axis, represented by a horizontal bar with an equal length to that of the peptide. The position of the bar along the vertical axis marks the fraction exchanged after 10 sec. The y-axis error bars are standard deviations calculated from three independent experiments. The coloured rectangles correspond to groups of peptides with different exchange rates (I-VII). (B), (D) 3D models of DmChd64 and TcChd64, respectively. The putative structures were generated using the I-TASSER web-based tool [20] using sequences of recombinant DmChd64 and TcChd64 as described previously [8] and the results were visualised using PyMOL [30]. The coloured regions correspond to groups of peptides with different exchange rates (I-VII): region I (DmChd64 1–39, TcChd64 1–27; blue), region II (DmChd64: 40–50, TcChd64: 28–45; turquoise), region III (DmChd64: 46–65, TcChd64: 44–53; grey), region IV (DmChd64: 66–74, TcChd64: 54–63; green), region V (DmChd64: 76–101, TcChd64: 63–89; yellow), region VI (DmChd64: 98–135, TcChd64 90–122; orange), region VII (DmChd64: 136–211, TcChd64 123–199; red). The arrows point to the W residues, marked in black.

Disordered regions of native DmChd64 and TcChd64 were mapped via HDX-MS. HDX exploits variations in the chemical exchange rate of backbone amide hydrogens in proteins. Hydrogens that are deeply buried in the protein core or that form hydrogen bonds only exchange if the protein undergoes a conformational change. Regions of ID exchange rapidly, and the exchange is completed after only a few seconds of exposure to D_2_O buffers [19]. To better visualise the HDX results (Fig 1A and 1C), we generated 3D models of the putative structures of DmChd64 and TcChd64 using I-TASSER [20]. The different regions of amide hydrogen exchange were superimposed on the I-TASSER model via colour coding (Fig 1B and 1D). DmChd64 and TcChd64 are homologues with 74% sequence identity. Recombinant DmChd64 and TcChd64 contain additional amino acids from affinity tags, which facilitate their purification [8]. As shown in Fig 1A and 1C, fragments in both Chd64 proteins rapidly exchange after 10 seconds of incubation in D_2_O buffer. Peptides covering the first 39 residues in the N-terminal region in *Drosophila* and 27 residues in *Tribolium* Chd64 exchange almost all of the amide hydrogens. This denotes high fragment flexibility due to the presence of ID (Fig 1A–1D, region I, marked in blue). Additionally, the presence of a larger region with less frequent exchange indicates well-ordered structure. This region comprises residues 40–139 in *Drosophila* and 28–122 in *Tribolium* and most likely corresponds to the CH domain, which is a well-defined protein module. Proteins containing the CH domain are abundant in all metazoans and are involved in actin binding and signal transduction [7]. An X-ray of the CH domain of human spectrin reveals its well-ordered helical structure [6]. Interestingly, the pepsin digestion patterns in the central regions of DmChd64 and TcChd64 are very similar. These globular fragments contain five distinct regions with significantly varied exchange rates. Consequently, a helix and loops in the CH domain from both Chd64 proteins are predicted to exist based on the crystallographic structure of the human spectrin CH domain. Pepsin cleavage generated a series of short peptides (Fig 1A and 1C, region II) at the beginning of the globular module between residues 40–50 and 28–45 in *Drosophila* and *Tribolium*, respectively, where the exchange rate was nearly zero, corresponding to a stable helical fragment (Fig 1B–1D, marked in turquois). The next series included peptides in which the exchange rate was increased, comprises residues 46–65 in *Drosophila* and 44–53 in *Tribolium* (Fig 1A and 1C, region III). These peptides mainly correspond to a loop (Fig 1B and 1D, marked in grey). However, another stable, strongly protected helical region was identified in residues 66–74 and 54–63 of DmChd64 and TcChd64, respectively (Fig 1A–1D, region IV, marked in green). In residues 76–101 of *Drosophila* and 63–89 of *Tribolium*, deuteration occurred at a rate of 60–80% (Fig 1A and 1D, region V), indicating the presence of a moderately flexible region, corresponding to a long loop in the I-TASSER model (Fig 1B and 1D, marked in yellow). The last distinct region within the globular core, located between residues 98–135 in DmChd64 and 90–122 in TcChd64, was also more protected (Fig 1A and 1C, region VI) and corresponded to a helix probably located along the edge of the CH domain (Fig 1B and 1D, marked in orange). Peptides covering the C-termini underwent rapid exchange, and deuteration was completed after 10 s of exposure to D_2_O buffer (Fig 1A–1D, region VII, marked in red). These regions, beginning at residue 130 in DmChd64 and 125 in TcChd64, are disordered. Overall ID accounts for 47% of the DmChd64 and 49% of the TcChd64 sequence.

Considering all of the above results, DmChd64 and TcChd64 possess a dual structural nature: their globular core appears to be outflanked by IDRs, and the extent of ID is significant. In both wild-type Chd64 proteins, the N-terminal IDR comprises 25 amino acid residues, whereas the C-terminal IDR comprises approximately 75 residues. Thus, approximately half of each polypeptide chain is disordered.

### Effects of GdmCl on the secondary structures by Far-UV CD spectroscopy

**Fig 2:**
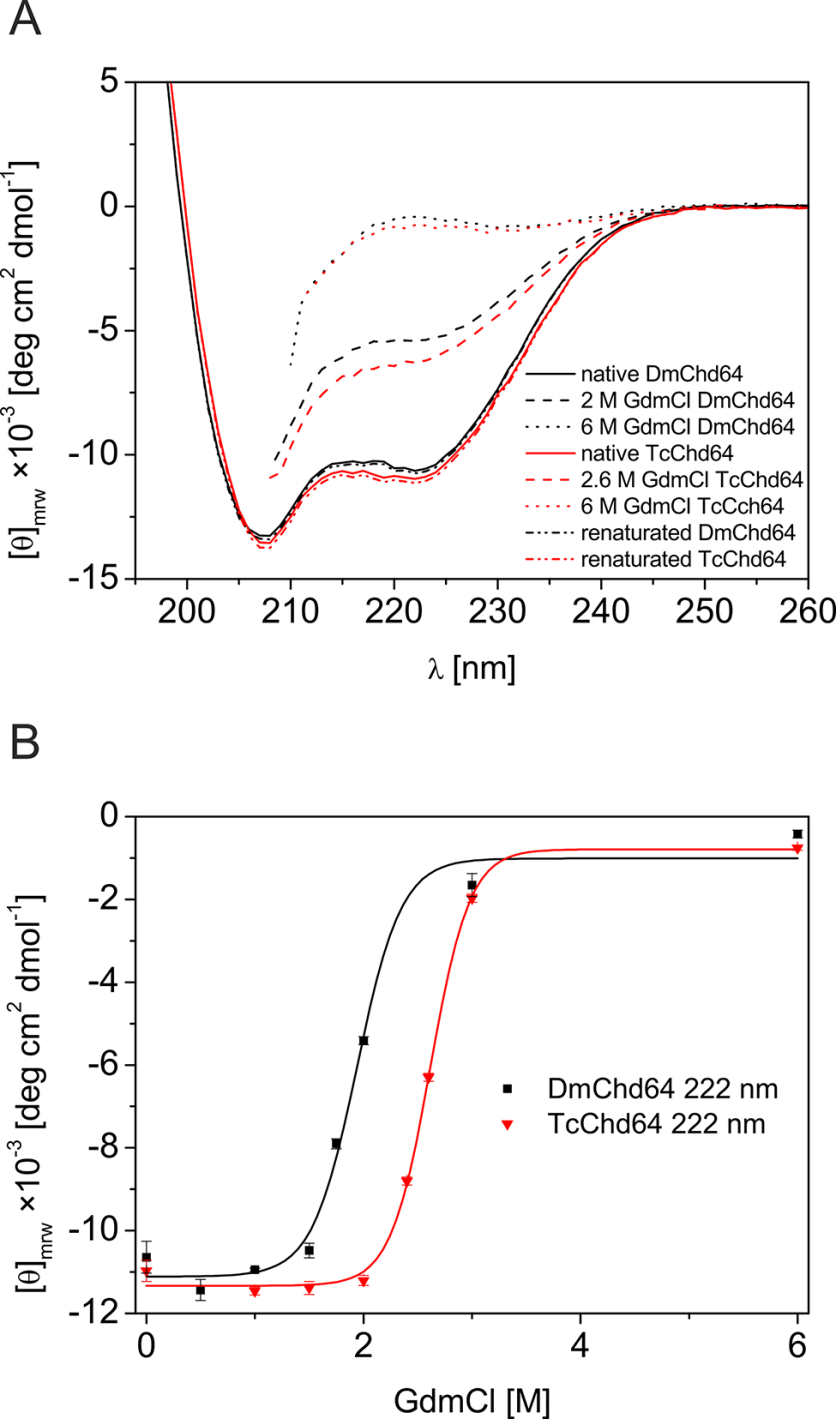
Study of DmCh64 and TcChd64 examination under various conditions by far-UV CD. (A), (B) The effect of GdmCl on the secondary structures of DmCh64 and TcChd64. (A): Representative CD spectra of DmChd64 (black) and TcChd64 (red) examined at different GdmCl concentrations. The spectra of renatured proteins, recorded in buffer A are presented by black dash–dot line for DmChd64 and red dash–dot line for TcChd64. The spectra were recorded in buffer A at 20°C under native (solid line) or denaturing conditions in the presence of 2.0 M or 6.0 M and 2.6 M or 6.0 M GdmCl (dashed lines) for DmChd64 and TcChd64, respectively. (B) *θ*_222_ of DmChd64 (black squares) and TcChd64 (red triangles) as a function of the GdmCl concentration.

HDX revealed that DmChd64 and TcChd64 have a dual structural nature. Based on this finding, we investigated the unfolding process to gain more insight into the molecular properties of Chd64 molecules. Changes in the secondary structural content and the tertiary structural integrity at various stages of the unfolding process were recorded using far-UV CD spectroscopy, fluorescence spectroscopy and SEC. Far-UV CD spectroscopy was employed first to monitor changes in the secondary structural content in relation to increasing GdmCl concentrations. The obtained spectra were compared with native Chd64 spectra. Our chemical denaturation studies revealed that DmChd64 is less resistant to action of the denaturant compared with TcChd64. As shown in Fig 2A, the DmChd64 signal in 2.0 M GdmCl decreased by approximately half, whereas 2.6 M GdmCl was required to observe a similar effect with TcChd64. For both proteins, the ellipticity measured by far-UV CD spectroscopy approached zero at 6.0 M GdmCl, indicating complete destabilisation of the ordered secondary structure of Chd64. To elucidate the nature of the native-to-unfolded transition, we plotted the ellipticity at 222 nm against the increasing GdmCl concentration (Fig 2B). Once again, TcChd64 shows greater stability in the presence of GdmCl, as unfolding occurs at higher GdmCl concentrations compared with those required for DmChd64 to unfold. The midpoint (Cm) of the native-to-unfolded transition was 1.94 ± 0.10 M GdmCl and 2.57 ± 0.12 M GdmCl for DmChd64 and TcChd64, respectively (Table 1). The sigmoidal shape and high steepness of the transition curve are typical of globular proteins undergoing native-to-unfolded transitions. These findings are in agreement with the HDX results presented above, denoting the presence of a well-ordered core in both proteins. The far-UV CD spectroscopy results revealed cooperative native-to-unfolded transitions and high resistance of ordered secondary structures in both Chd64 proteins to the action of the denaturant.

**Table 1:** Unfolding parameters of chemically denatured DmChd64 and TcChd64.

	DmChd64			TcChd64		
	Far UV CD	Fluorescence	SEC	Far UV CD	Fluorescence	SEC
**C**_**m**_ **[M]**	1.94±0.10	1.32±0.03	2.09±0.08	2.57±0.12	1.33±0.04	2.57±0.10

± standard deviation were calculated from three independent experiments.

### Chd64 tertiary structure destabilisation studied by fluorescence spectroscopy

**Fig 3:**
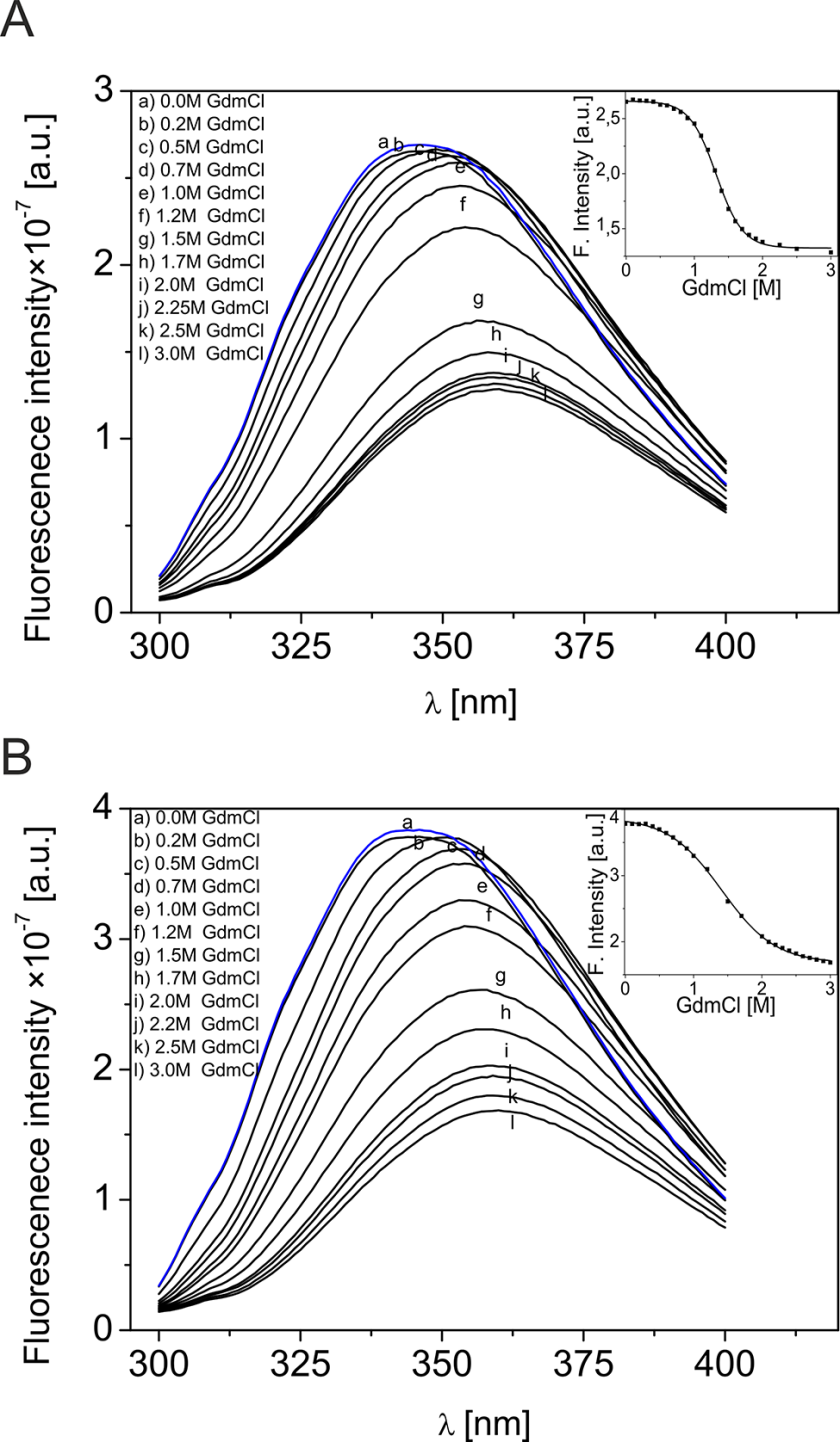
Fluorescence analysis of the effect of GdmCl on DmChd64 and TcChd64. Fluorescence emission spectra (*λ*_*ex*_ = 280 nm) of DmChd64 (A) and TcChd64 (B) in the presence of different GdmCl concentrations (a-l). The spectra were recorded between 300 and 400 nm under native (a) or denaturing conditions in the presence of 0.2 M (b), 0.5 M (c), 0.7 M (d), 1.0 M (e), 1.2 M (f), 1.5 M (g), 1.7 M (h), 2.0 M (i), 2.2 M (j), 2.5 M (k), 3.0 M (l) GdmCl. The blue lines represent spectra of proteins recorded after renaturation. Inset: changes in fluorescence intensity monitored at 346 nm for DmChd64 (A) and TcChd64 (B) titrated with GdmCl. Data points were taken from the fluorescence spectra.

To investigate the influence of GdmCl on the tertiary structures of DmChd64 and TcChd64, changes in the fluorescence of tryptophan residues were studied. Wild-type DmChd64 and TcChd64 have two tryptophan (W) residues within the CH domain (W34, W114). In the I-TASSER model (Fig 1B and 1D), W34 is buried within the core, whereas W114 exhibits good accessibility to the solvent; thus, the maximum fluorescence of native proteins is maintained at 346 nm. GdmCl exhibited a similar denaturing effect on the tertiary structures of DmChd64 and TcChd64. Increasing GdmCl concentrations resulted in opening of the folded core and exposure of the tryptophan residues to the solvent; these changes were observed as a red shift from 346 to 360 nm with 52% and 55% drops in intensity for DmCh64 and TcChd64, respectively (Fig 3A and 3B). These results indicate that GdmCl exposed tryptophan residues to the solvent and destabilised the tertiary integrity of proteins. As presented in Fig 3A and 3B, the transition curves corresponding to DmChd64 and TcChd64, respectively, have sigmoidal shapes, indicating the occurrence of cooperative native-to-unfolded transition. The transition Cm values from the fluorescence experiments were 1.32 ± 0.03 M GdmCl and 1.33 ± 0.04 M GdmCl for DmChd64 and TcChd64, respectively. Interestingly, the unfolding experiment monitored by far-UV CD spectroscopy (discussed above) showed that GdmCl at approximately 1.3 M did not significantly impair the secondary structure of Chd64. The secondary structural content at the transition Cm of the fluorescence experiments is comparable to that in native Chd64. Thus, helices formed in the globular core are largely preserved during Chd64 denaturation, although some elements of the tertiary structure probed by tryptophan residues are partially destabilised.

### Look at the structural changes in the entire protein molecule induced by GdmCl

**Fig 4:**
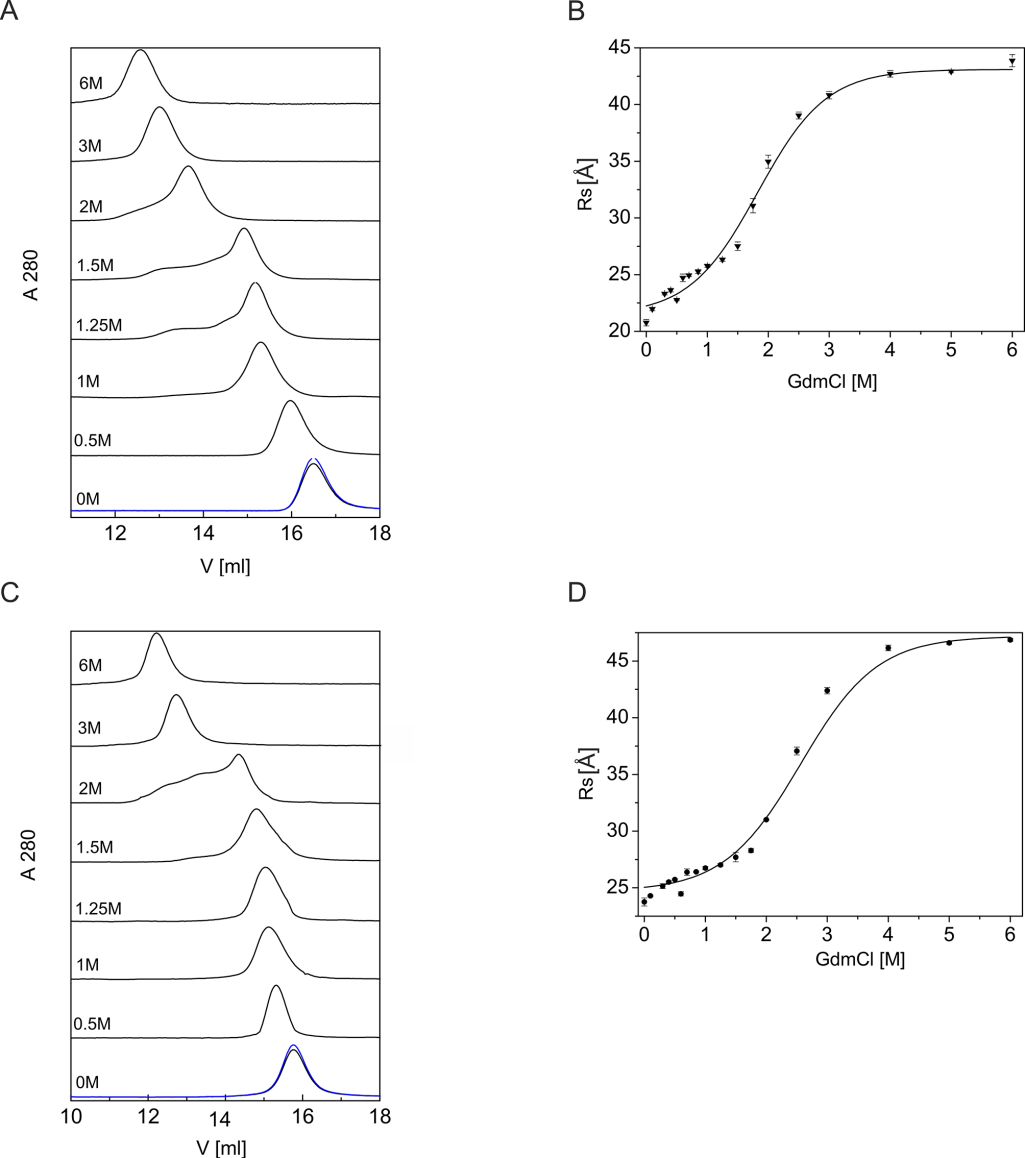
Changes in the hydrodynamic properties of DmChd64 and TcChd64 induced by GdmCl. (A), (C) SEC analysis of DmChd64 (A) and TcChd64 (B) in the presence of GdmCl. The curves represent profiles of DmChd64 and TcChd64 chromatographed at different GdmCl concentrations. The blue lines represent elution peaks of renatured proteins chromatographed in buffer A. Purified DmChd64 and TcChd64 (0.5 mg/ml in buffer A with an appropriate GdmCl concentration) were injected into a Superdex 200 10/300 GL column equilibrated with buffer A of the same denaturant concentration for each sample at room temperature, with a flow rate of 0.5 ml/min. (B), (D) Using the calibration curve (see Materials and Methods) and the elution volumes determined in (A) and (C), the R_s_ values of DmCh64 (B) and TcChd64 (D) were calculated and plotted against the GdmCl concentration.

We used SEC to investigate the structural changes that occur during the native-to-unfolded transition of the protein molecules. Using SEC, molecules with different levels of compactness are separated [21]. Previously, we found that the R_s_ values of DmChd64 and TcChd64 are larger than the theoretical values [8]. Consequently, the molecular volumes are also larger compared with globular equivalents of the same theoretical mass. We correlated this with the existence of a putative partially disordered conformation demonstrated by HDX. Increasing GdmCl concentrations strongly affected the R_s_ of Chd64. Notably, at 1.25 M and 1.50 M GdmCl for DmChd64 and TcChd64, respectively, the elution peaks become broader and asymmetrical (Fig 4A and 4C) indicating that both Chd64 proteins undergo significant conformational changes and that Chd64 protein populations become heterogeneous during the native-to-unfolded transition. Some additional states exist that are neither native nor unfolded (Fig 4A, 1.25–2 M GdmCl and 4B, 1.5–2 M GdmCl). The appearance of such discrete conformational states suggests that some conditions alter the shapes of molecules, indicating that Chd64 is a relatively flexible, pliable molecule that can exist in different conformational states. In a buffer containing 3.0–6.0 M GdmCl, the hydrodynamic volume of DmChd64 significantly increased and peaks became symmetrical again, suggesting that the equilibrium between the native and unfolded forms shifted towards the latter from (Fig 4A). A similar pattern was observed for TcChd64 (Fig 4C), although the respective transition occurred at higher GdmCl concentrations. At 1.25 M GdmCl, at which point DmChd64 was undergoing conformational changes and existed in different conformational forms (Fig 4A), TcChd64 still generated a symmetrical peak (Fig 4C). However, different conformers of TcChd64 were present at 1.5 M GdmCl. At 3.0 M GdmCl, the elution volume decreased, indicating that TcChd64 was already denatured. The apparent R_s_ increased twice during the transition between the native and denatured forms, reaching 22.76 ± 0.68 Å for native DmChd64 to 43.83 ± 0.90 Å for denatured proteins at 6.0 M GdmCl. The R_s_ of TcChd64 increased from 23.75 ± 0.71 Å for native proteins to 46.87 ± 0.92 Å for proteins in 6 M GdmCl. These GdmCl-induced unfolding study results revealed that the native-to-unfolded transition is a complex process for both Chd64 proteins. Fluorescence spectroscopy results showed that 1.3 M of GdmCl induced destabilisation of the local tryptophan residue environment (Fig 3A and 3B). This, however, does not significantly influence the global architecture of proteins that depends on secondary structural elements. At 1.3 M, these elements were primarily preserved in both Chd64 proteins; however, the population of Chd64 molecules became heterogeneous (Fig 4A and 4C), indicating that both Chd64 proteins could adopt distinct conformational states under certain conditions.

### The intermediate state of the native-to-unfolded transition

**Fig 5:**
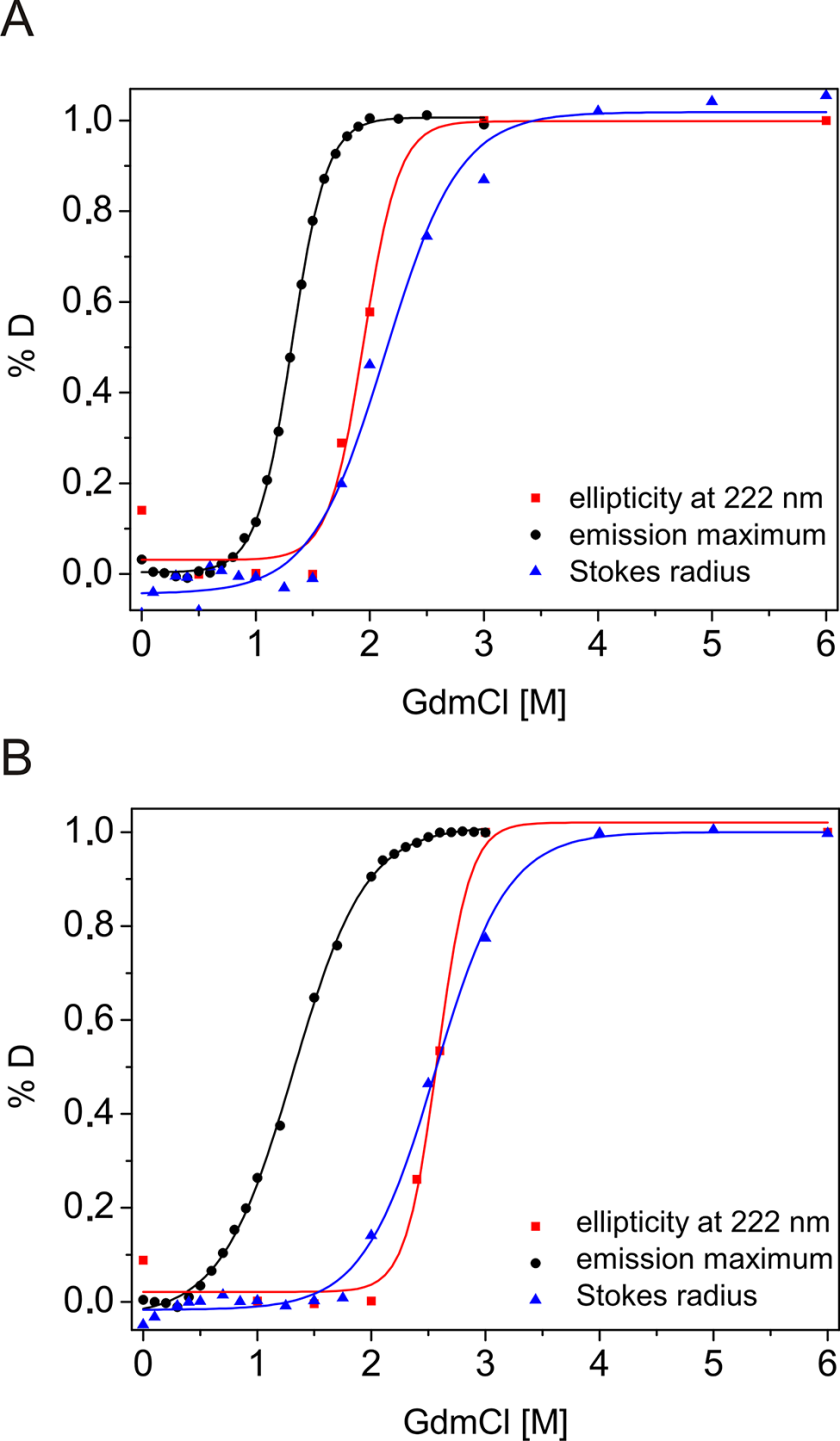
GdmCl induced conformational changes in DmChd64 and TcChd64. GdmCl-mediated transitions of DmChd64 (A) and TcChd64 (B) unfolding, monitored by far-UV CD (red squares), fluorescence spectroscopy (black dots) and SEC (blue triangles). Normalized transition curves were calculated using Eq 3. The calculated transition midpoints (C_m_) are presented in Table 1.

**Fig 6:**
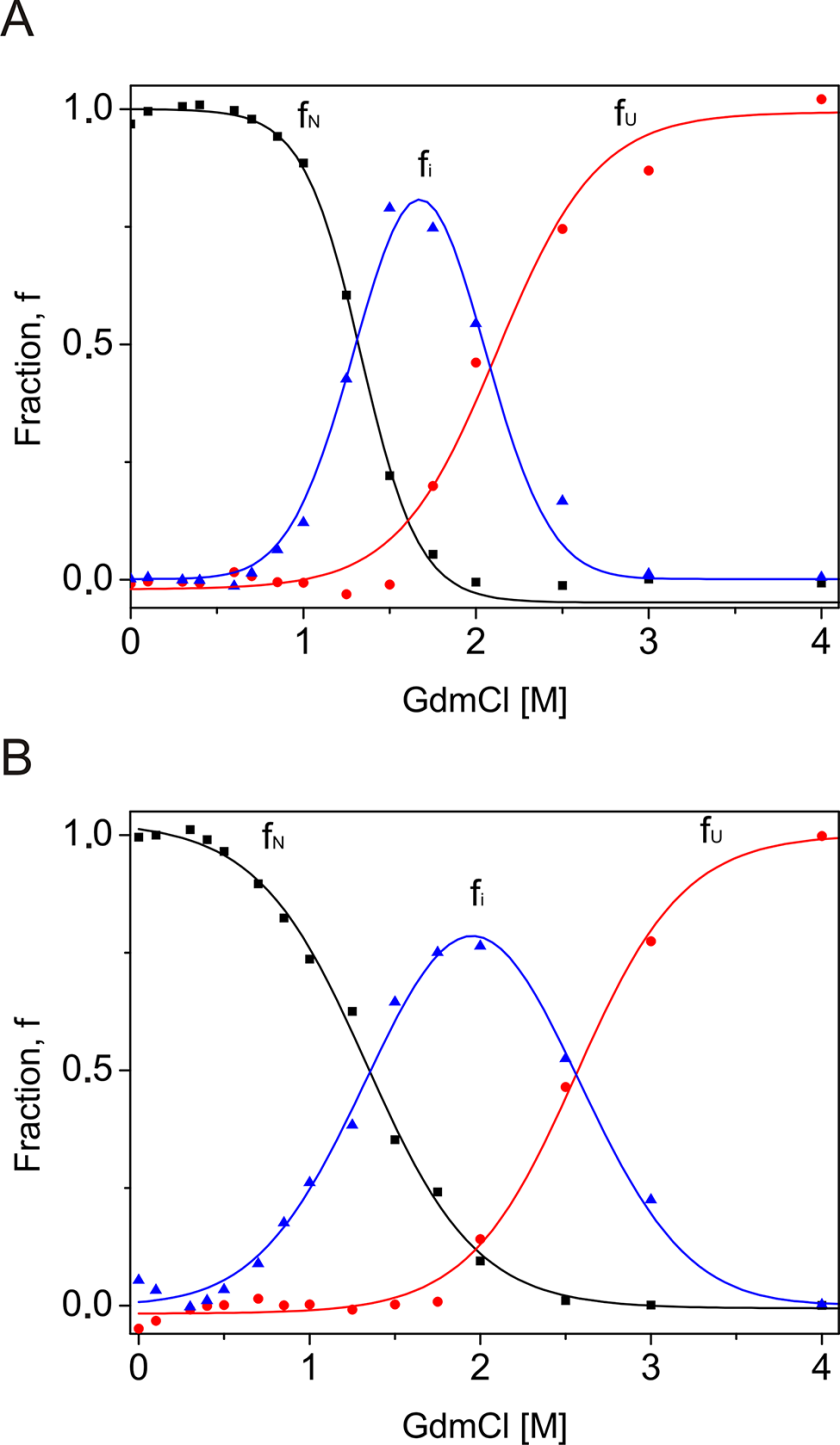
The three-state transition of DmChd64 and TcChd64 at different GdmCl concentrations. Populations of native, unfolded and intermediate molecules for DmChd64 (A) and TcChd64 (B) were expressed as a fraction of all DmChd64 and TcChd64 molecules. The fraction of native (*f*_*N*_) molecules (black squares) was calculated from the fluorescence measurements, using Eq 4, and the fraction of unfolded (*f*_*U*_) molecules (red dots) was calculated from SEC and far-UV CD analyses, using Eq 5. In our case, *f*_*N*_ + *f*_*U*_ < 1 and the intermediate state fraction (*f*_*i*_) (blue triangles) was defined by Eq 6.

DmChd64 and TcChd64 unfolding, as measured using a range of techniques, is reversible and complex. Molecules in certain conditions can exist in a well-defined intermediate conformation. Far-UV CD and fluorescence spectroscopy and SEC data were used to calculate populations of molecules under particular states. The percentage of molecules after conformational transition (%D) was plotted against increasing GdmCl concentrations (Fig 5A and 5B), and the transition Cm value for each experiment was calculated (Table 1). The unfolding curves did not overlap. For both DmChd64 and TcChd64, significant differences were found between the fluorescence spectroscopy results and the far-UV CD spectroscopy and SEC results. The fluorescence measurements, which provide insight into protein integrity, revealed that the transition occurred from 0.8–2.0 M GdmCl for DmChd64 and 0.5–2.5 M for TcChd64, with a transition Cm of 1.32 ± 0.03 M and 1.33 ± 0.04 M GdmCl for DmChd64 and TcChd64, respectively (Fig 5A and 5B). The far-UV CD results revealed changes in the secondary structural content. The transition occurred at higher GdmCl concentrations compared with those found by fluorescence. For DmChd64, the transition occurred at 1.4–2.2 M GdmCl, with a Cm of 1.94 ± 0.10 M GdmCl; for TcChd64, the transition occurred at 2.0–3.0 M, with a Cm of 2.57 ± 0.12 M GdmCl (Fig 5A and 5B). Additionally, the transition occurred at higher denaturant concentrations when monitored by SEC, falling within a range of 1.0–3.0 M and 1.5–3.5 M GdmCl, with a Cm of 2.09 ± 0.08 M and 2.57 ± 0.10 M GdmCl, for DmChd64 and TcChd64, respectively. These values indicate that unfolding processes begin with destabilisation of the molecular integrity, followed by the unwinding of secondary structures. Between these two phases, Chd64 exists as an intermediate state population (Fig 6A and 6B) that does not exceed 1.7 M GdmCl for DmChd64 and 1.9 M GdmCl for TcChd64. The intermediate state content is significant and can comprise 75% of the entire population of molecules. Consequently, GdmCl-induced Chd64 unfolding can be defined by a three-state model that includes native, intermediate and unfolded states. In the intermediate state, which resembles a MG conformation, helices formed in the globular core are largely preserved but are not fixed in 3D space [22,23].

## Discussion

**Fig 7:**
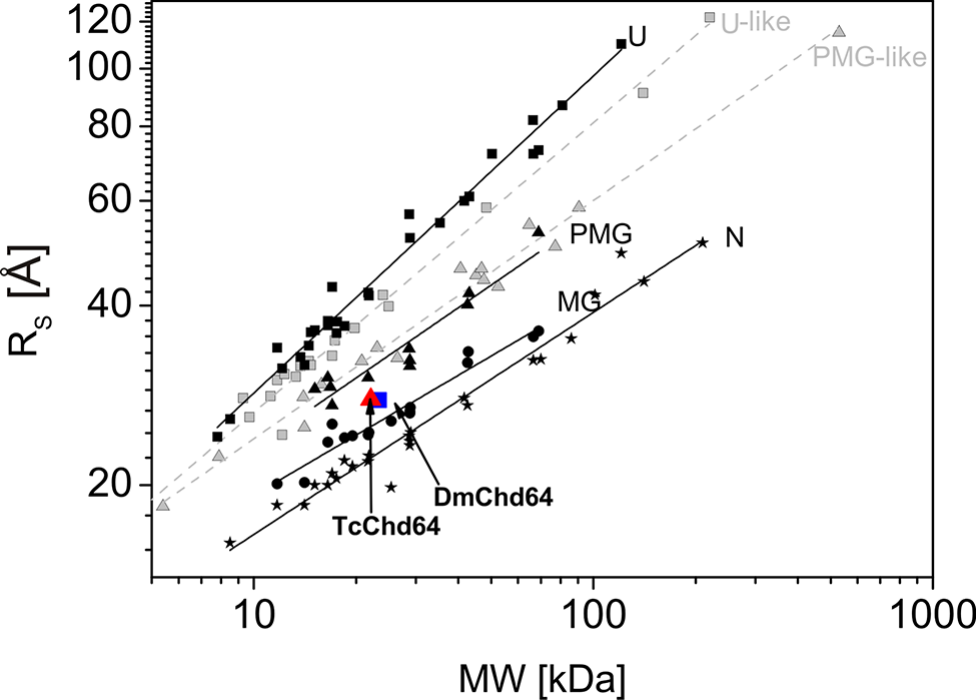
The Stokes radius (R_s_) versus relative molecular mass (MW) plot [22]. The dependence of Stokes radius (R_s_) values on the relative molecular masses (MW) of the four equilibrium states of globular proteins (solid lines) and two subclasses of IDPs (dashed lines). The globular proteins are marked in black: native (N, stars), molten globules (MGs, dots), pre-molten globules (PMGs, triangles), and 6 M GdmCl-unfolded proteins (U, squares). The IDPs are marked in grey: U-like (squares) and PMG-like (triangles). The data for globular proteins were drawn from [31], and the data for the IDPs were drawn from [22]. The values for DmChd64 and TcChd64 in the intermediate state are represented as blue squares and red triangles, respectively.

The structures of *Drosophila melanogaster* and *Tribolium castaneum* Chd64 proteins are characterised by a heterogeneous nature. Our HDX experiments revealed that their N- and C-termini are disordered, whereas their central regions comprise a well-ordered globular core. This globular fragment corresponds to the CH domain. Most CH domain-containing proteins are involved in actin cross-linking and bundling, although some modulate cellular signalling [7,24]. Li *et al*. [4] suggested that in *D*. *melanogaster*, Chd64 is a key component of a multiprotein complex that forms on JHRE when JH level is high. Other components of the complex include EcR, Usp, FKBP39 and Met, a potential JH receptor. The mechanism that governs the multiple interactions between Chd64, JHRE and the above-described binding partners remains unknown. Disordered tails have considerable potential to participate in multiple interactions. Generally speaking, disorderness is not equally allocated- within protein sequences. Most often it is present in the termini of the protein chains [25]. The differences in amino acid composition and the length of the disordered termini from different protein families points toward their functional importance [revised in 25]. In addition to mapping the disordered region, we studied the nature of the native-to-unfolded transitions of DmChd64 and TcChd64, both of which have a dual-natured, heterogeneous structure. Chd64 is a relatively small (approx. 21 kDa) single-domain protein. Our HDX experiment showed that nearly half of the proteins were disordered and that IDRs outflanked the stable globular core. Studies of the nature of native-to-unfolded transitions in proteins with similar to Chd64, dual nature are limited. Typically, urea and GdmCl-induced unfolding of globular protein is described using a two-state model [26], which assumes that an intermediate equilibrium state does not exist between the native and unfolded species [27]. Some proteins, however, unfold through an intermediate state [28]. Populations of molecules in the intermediate state may have pronounced secondary structures but no rigid tertiary conformation or in the case of enzymes, no activity. These molecules have a loosely packed core and fluctuate much more drastically than their native counterparts. This intermediate state was defined as the MG state [29]. Our results show that DmChd64 and TcChd64 undergo a three-state transition with a well-defined intermediate state (Fig 6A and 6B). The transition, as assessed by fluorescence spectroscopy, occurred at lower GdmCl concentrations (Table 1). Both proteins contain two tryptophan residues buried within the globular core. Their positioning within the primary structures of wild type DmChd64 and TcChd64 is identical (i.e., W34 and W114) to their positioning in the predicted 3D structure (Fig 1B and 1D). According to the model, W34 is found near the edge of the helix and is not buried deep within the core, whereas W114 is located in the middle of the helix and is less exposed to solvents. Consequently, the maximum fluorescent emission spectrum value of native DmChd64 and TcChd64 occurs at 346 nm, indicating partial exposure of the tryptophan residues to the solvent. GdmCl causes the globular structure to open, exposing the tryptophan residues to the polar solvent as part of a bathochromic shift in the fluorescence emission intensity (Fig 3A and 3B). Notably, in contrast to the far-UV CD and SEC results, the Cm values for DmChd64 and TcChd64 determined by fluorescence are comparable, suggesting that differences in the stability of both Chd64 proteins are observed as changes in the secondary structural content and that changes in hydrodynamic properties do not originate from regions surrounding tryptophan residues. Indeed, our SEC experiments revealed that the hydrodynamic dimension increases in concentrations corresponding to the fluorescence transition Cm. At 1.25 M GdmCl for DmChd64 and 1.5 M for TcChd64, the elution peak was no longer symmetrical and became broader. This broadening of elution peaks in the middle of the native-to-unfolded transition may reflect the presence of an intermediate state. Here, partially unfolded populations and molecules in their native conformation meet. Moreover, at 1.25–1.5 M GdmCl for *Drosophila* and 1.5–2.0 M for *Tribolium*, additional peaks of lower elution volumes are observed (Fig 4A and 4C), indicating that Chd64 can adopt different shapes and exist in discrete conformations. This finding may explain its potential ability to form a multiprotein complex that is involved in regulating hormonal signal transduction. The highest concentrations of molecules in intermediate conformations were found between the Cm values calculated by fluorescence emission experiments and by both far-UV CD and SEC (Fig 6A and 6B). This finding indicates that molecules in their intermediate state have no tertiary integrity but that simultaneously, their secondary structural contents are mostly preserved. To classify the structural conformation, we compared the R_s_ values of molecules at 1.7 M GdmCl for DmChd64 and 1.9 M GdmCl for TcChd64 against the corresponding theoretical molecular masses. The R_S_-versus-relative molecular mass plot revealed that both Chd64 proteins were located in the MG area (Fig 7). At 2.0 M GdmCl for DmChd64 and 2.5 M GdmCl for TcChd64, the MG population began to decrease. To conclude the unfolding process, proteins were fully destabilised, secondary structures unwound, and coil-like conformers appeared.

In summary, the two homologous proteins DmChd64 and TcChd64 exhibit similar structural organisation. Both proteins have disordered tails that outflank a globular core. The native-to-unfolded transition of Chd64 is a three-state process that involves the formation of a well-defined intermediate state population. In their intermediate state, molecules show MG characteristics. Importantly, DmChd64 and TcChd64 are pliable and can adopt different conformational states. The plasticity of these proteins and the existence of terminal IDRs with their innate flexibility and adaptability, may serve as a platform for multiple interactions, possibly allowing for the regulatory functions of Chd64 in insect hormonal signalling.
